# Production of a Promising Biosynthetic Self‐Assembled Nanoconjugate Vaccine against *Klebsiella Pneumoniae* Serotype O2 in a General *Escherichia Coli* Host

**DOI:** 10.1002/advs.202100549

**Published:** 2021-05-24

**Authors:** Zhehui Peng, Jun Wu, Kangfeng Wang, Xin Li, Peng Sun, Lulu Zhang, Jing Huang, Yan Liu, Xiaoting Hua, Yunsong Yu, Chao Pan, Hengliang Wang, Li Zhu

**Affiliations:** ^1^ State Key Laboratory of Pathogen and Biosecurity Beijing Institute of Biotechnology No. 20, Dongda Street, Fengtai District Beijing 100071 P. R. China; ^2^ Department of Infectious Diseases Sir Run Run Shaw Hospital College of Medicine Zhejiang University 866 Yuhangtang Rd Hangzhou 310058 P. R. China

**Keywords:** conjugate vaccine, *Klebsiella pneumoniae*, nanovaccine, self‐assembly

## Abstract

*Klebsiella pneumoniae* has emerged as a severe opportunistic pathogen with multiple drug resistances. Finding effective vaccines against this pathogen is urgent. Although O‐polysaccharides (OPS) of K. pneumoniae are suitable antigens for the preparation of vaccines given their low levels of diversity, the low immunogenicity (especially serotype O2) limit their application. In this study, a general *Escherichia coli* host system is developed to produce a nanoscale conjugate vaccine against *K. pneumoniae* using the Nano‐B5 self‐assembly platform. The experimental data illustrate that this nanoconjugate vaccine can induce an efficient humoral immune response in draining lymph nodes (dLNs) and elicit high titers of the IgG antibody against bacterial lipopolysaccharide (LPS). The ideal prophylactic effects of these nanoconjugate vaccines are further demonstrated in mouse models of both systemic and pulmonary infection. These results demonstrate that OPS with low immunogenicity can be changed into an effective antigen, indicating that other haptens may be applicable to this strategy in the future. To the knowledge, this is the first study to produce biosynthetic nanoconjugate vaccines against *K. pneumoniae* in *E. coli*, and this strategy can be applied to the development of other vaccines against pathogenic bacteria.

## Graphical abstract

A general *E. coli* host system is developed to produce nanoscale conjugate vaccines against pathogens such as *K. pneumoniae* with low immunogenicity O‐polysaccharides. Such nanovaccine can be well delivered to lymph nodes and induced excellent humoral immune stimulation effect, and induced strong prophylactic effects against MDR *K. pneumoniae* in systemic and pulmonary‐infection models.

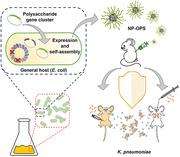

## 1 Introduction

*Klebsiella pneumoniae* is the causative agent of many infectious diseases, including but not limited to pneumonia, bacteremia, sepsis, urinary‐tract infection, meningitis, and pyogenic liver abscess. It is reported to be the most common multidrug‐resistant (MDR) species (accounting for 35.2% of such species) and has significantly increased (by 16.4%) worldwide.^[^ ^1^ ^]^ In particular, expression of carbapenemase gives carbapenem‐resistant *K. pneumoniae* (CRKP) significantly higher rates of morbidity and mortality than the classic bacterium.^[^ ^2^ ^]^

Vaccines play an important role in the prevention and control of infectious diseases. Although discovery of an effective vaccine against *K. pneumoniae* is very urgent at present, there is still no product approved in the market. Various vaccines are under development. An inactivated whole‐cell vaccine, the first vaccine against *K. pneumoniae*, was developed in 1970,^[^ ^3^ ^]^ but this kind of vaccine has not been used clinically due to its poor safety level. Subsequent technologies have elaborated to create protein‐ or polysaccharide‐based vaccines such as outer‐membrane protein (OMP),^[^ ^4^ ^]^ fimbriae protein,^[^ ^5^ ^]^ and surface polysaccharide vaccines.^[^ ^6^ ^]^ Although safety and effectiveness have improved, the low immunogenicity of most antigens results in a weak level of protection, meaning that new strategies to boost antigen response are required. To date, glycoconjugate vaccines, which couple bacterial polysaccharides with carrier proteins, have been among the most successful bacterial vaccines due to their ability to induce both T‐cell and B‐cell immune response and long‐term protection.^[^ ^7^ ^]^ Linking polysaccharides with protein components could convert them from T‐cell‐independent into T‐cell‐dependent antigens. Some studies have proven the effectiveness of polysaccharides as antigens in *K. pneumoniae*.^[^ ^8^ ^]^ Therefore, conjugate vaccine is a promising strategy for *Klebsiella* prevention.

However, more than 77 capsular polysaccharide (CPS) serotypes of *K. pneumoniae* have been found, and 25 of them comprise almost 70% of clinical isolates.^[^ ^9^ ^]^ Excessive capsular serotypes make it difficult to prepare multivalent vaccines for wide protection against *K. pneumoniae*, limiting the development and application. By contrast, another type of surface polysaccharide, O‐polysaccharides (OPS) has only 8 serotypes. About 80% of clinical isolates belong to one of four serotypes (O1, O2, O3, and O5), making it an ideal antigen for conjugate vaccines.^[^ ^10^ ^]^ Particularly, researchers also found that serotype O2 has selective advantages under antibiotic pressure, and it is becoming the predominant serotype in the isolated extended‐spectrum beta‐lactamase (ESBL; 35%) and carbapenem‐resistant *Enterobacteriaceae* (CRE; 50%) subgroups of *K. pneumoniae*.^[^ ^11^ ^]^ Therefore, a vaccine against O2 strains could provide better coverage against MDR bacterial isolates. However, the immunogenicity of OPS is generally lower than that of CPS, especially in O2 polysaccharide repeat units composed of two simple galactoses (Figure S1A, Supporting Information).^[^ ^12^ ^]^ The uncomplicated structure makes it difficult for O2 antigen to stimulate an effective immune response using traditional vaccine strategies.

The application of nanotechnology has to lead innovations in the field of vaccinology. As efficient delivery systems, nanomaterials can significantly improve immune response to antigens.^[^ ^13^ ^]^ Many nanomaterials, such as polymeric nanoparticles (NPs), inorganic NPs, and liposomes, have been developed and widely used for vaccine preparations.^[^ ^14^ ^]^ However, most of those reported so far are difficult to use in preventive bacterial vaccines due to their lack of safety and biocompatibility. To solve these problems, our group has developed a fully biosynthesized proteinaceous Nano‐B5 vaccine platform.^[^ ^15^ ^]^ Once we ascertained that the expressed protein monomers could self‐assemble into NPs in periplasm, we introduced a protein glycosylation system that coupled the bacterial polysaccharides with the NPs.

**Figure 1:**
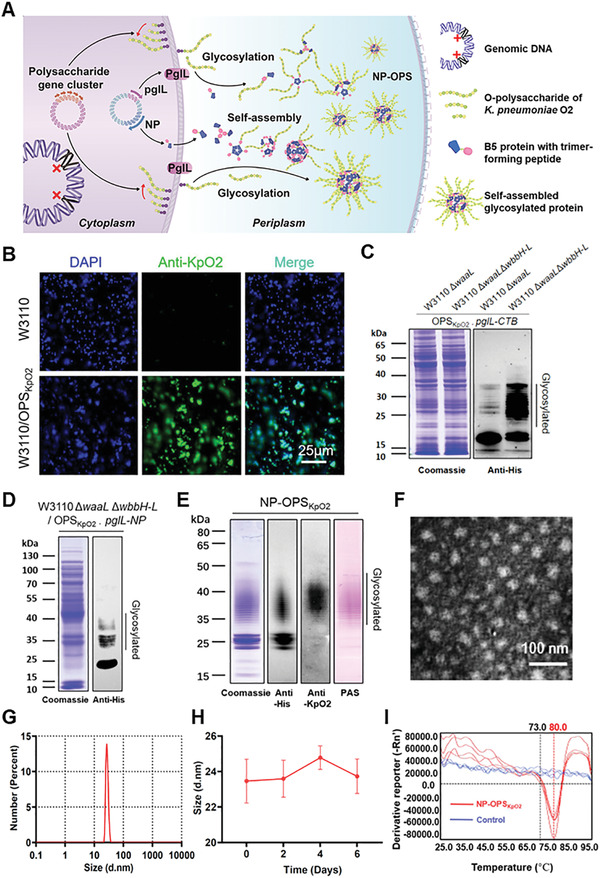
Expression and characterization of heterologous glycoprotein. A) Schematic diagram of *K. pneumoniae* nanoconjugate‐vaccine expression process in host cell. B) IF analysis of *K. pneumoniae* serotype O2 polysaccharide expression in strain W3110. C) Glycoproteins were detected in strains W3110△*waaL* and W3110△*waaL*△*wbbH‐L*. D) Western blot analysis of PglL‐mediated O‐linked nanoparticle glycosylation with antibodies against 6 × His Tag. E) Purified NP‐OPS_KpO2_ samples were separated by SDS‐PAGE and analyzed via Coomassie Blue staining, Western blot using antibody against 6 × His Tag and serum against *K. pneumoniae* serotype O2, and PAS. F,G) TEM image F) and DLS analysis G) of NP‐OPS_KpO2_. H) DLS analysis of NP‐OPS_KpO2_ size stability at different time points after filtering with 0.22 µm filter and incubation at 37 °C. I) Thermal stability of NP‐OPS_KpO2_ was assessed using a protein thermal‐shift assay with an increasing temperature of 25–95 °C

In this study, we applied this platform in the laboratory *E. coli* strain and synthesized a nanoconjugate vaccine that efficiently carried *K. pneumoniae* serotype O2 polysaccharide (**Figure** **1**A). Next, we proved that this nanoconjugate vaccine could be well delivered to lymph nodes and induced an excellent humoral immune stimulation effect. A series of animal experiments further demonstrated that this vaccine was safe and could induce strong prophylactic effects against MDR *K. pneumoniae* in lethal (or nonlethal) systemic or pulmonary‐infection models, with no need of aluminum hydroxide (Al) adjuvants.

## 2 Results

### 2.1 Biosynthesis of *K. Pneumoniae* Glycoconjugate Vaccine in Modified *E. Coli*

In previous studies, we successfully produced glycoconjugate vaccines in some attenuated pathogens.^[^ ^16^ ^]^ The next step required a general host with low‐cost, ease of culture propagation, and scalability. The laboratory *E. coli* strain became a possibility. After confirming the synthesis of heterologous *K. pneumoniae* serotype O2 polysaccharide in *E. coli* (Figure 1B; and Figure S1, Supporting Information), we hijacked this heterogeneous lipopolysaccharide (LPS) synthesis system to make bioconjugate vaccines bearing O2 polysaccharides by introducing the glycosyltransferase PglL, as we have demonstrated previously.^[^ ^16^ ^]^ However, a relatively low efficiency of protein glycosylation was observed in *E. coli* W3110△*waaL* strain (Figure S2, Supporting Information). To improve the production of glycoproteins, we further knocked out of the remaining OPS synthesis related genes (five genes from *wbbH* to *wbbL*), and named this modified host W3110△*waaL*△*wbbH‐L* (Figure S3, Supporting Information). After we introduced the expression vectors pACYC184‐OPS_KpO2_ (expression of O2 polysaccharide of *K. pneumoniae*) and pET28a‐*pglL*‐*CTB* (coexpression of glycosyltransferase PglL and cholera toxin B subunit [CTB] with glycosylation sequon) into the host bacteria W3110△*waaL*△*wbbH‐L*, the Western blot results of whole‐cell lysates showed that most of the carrier proteins CTB could be glycosylated (named C‐OPS_KpO2_), with an increase of over sevenfold (from 11.3% to 88.6%) in glycosylation efficiency by cumulative density (Figure 1C).

However, we found that the protection effect of the CTB‐based conjugate vaccines for O2 serotype of *K. pneumoniae* is not satisfactory as expected (Figure S4, Supporting Information),^[^ ^16^ ^]^ which might attribute to the weaker immunogenicity of its simple disaccharide repeat structure. In order to enhance immune response to this kind of polysaccharide antigens, a fully biosynthetic NP formed from proteinaceous monomers was used.^[^ ^15^ ^]^ The monomer of the NP contains CTB, a 4‐aa linker peptide (Gly‐Gly‐Ser‐Gly [GGSG]), a C‐terminal trimer‐forming peptide, and a 29‐aa glycosylation sequence (named 4573).^[^ ^15^ ^]^ The addition of the C‐terminal trimer‐forming peptide causes the bioconjugate to conglomerate into a quaternary spherical structure with protein at the core and O‐antigen polysaccharides extending at the periphery. After coexpression with PglL, NP, and *K. pneumoniae* O‐antigen gene cluster in W3110△*waaL*△*wbbH‐L*, OPS_KpO2_ were attached to NPs under catalysis of PglL in periplasm. As expected, the Western blot results showed the glycosylation bands of NP‐OPS_KpO2_ monomers located between 25 and 50 kDa (Figure 1D).

Then, we purified the glycoprotein and performed a series of characterizations. After separation by sodium dodecyl sulfate polyacrylamide gel electrophoresis (SDS‐PAGE), staining of SDS polyacrylamide gels with Coomassie Blue, periodic acid‐Schiff (PAS), and immunoblotting using anti‐6 × His Tag antibody and anti‐*K. pneumoniae* serotype O2 serum revealed the expected banding patterns for the NP‐OPS_KpO2_ monomers: the polysaccharide presented a typical ladder, and each rung thereof corresponded to a different repeat unit number (disaccharide; Figure 1E). A liquid chromatography tandem‐mass spectrometry (LC‐MS/MS) analysis revealed that the polysaccharide conjugated vaccine fabricated in *E. coli* bear the same OPS structure as that of *K. pneumoniae* serotype O2 strains (Figure S5, Supporting Information). Transmission electron microscopy (TEM) and dynamic light scattering (DLS) analyses of the purified extracts revealed that NP‐OPS_KpO2_ was about 25 nm in size (Figure 1F,G). The stability of the nanoscale glycoprotein was detected at different time points after incubation at 37 °C, and the particles could be kept stable after being stored for a long time (Figure 1H; and Figures S6 and S7, Supporting Information). Furthermore, we mixed the nanoconjugate vaccine with protein thermal‐shift dyes. When the temperature rose, those hydrophobic amino acid residues buried in the inner cavity of the NP were exposed to the dye and emitted more fluorescent signals. As shown (Figure 1I), the intensity of the signal began to increase at 73 °C, and melting temperature (*T*_m_) was established to be 80 °C, which meant that the nanoconjugate vaccine started to depolymerize at this temperature. This indicated a high temperature resistance.

### 2.2 Lymph Node Targeting and Immune Activation by the Nanoconjugate Vaccine

**Figure 2:**
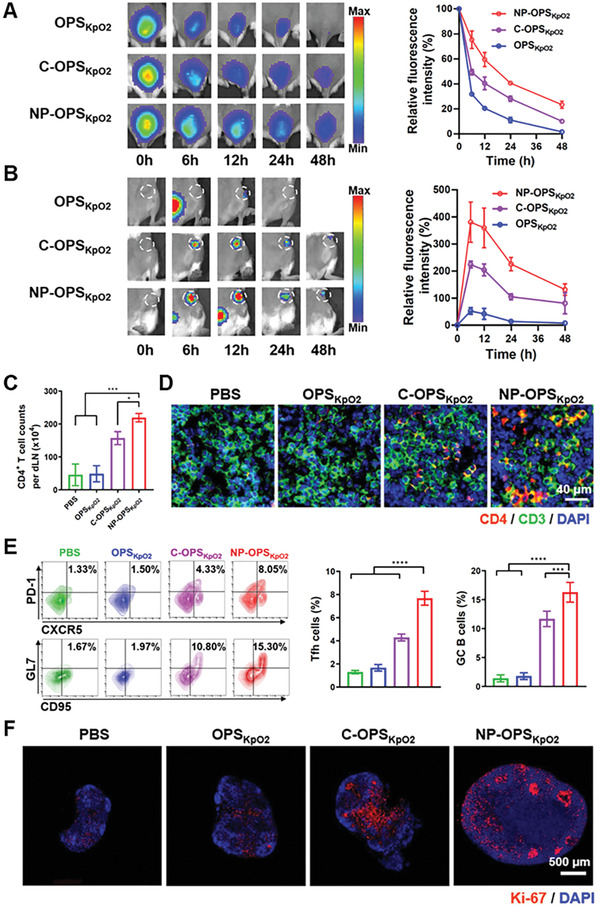
Lymph node targeting and immune activation by nanovaccine. A,B) Representative images and corresponding quantitative fluorescence analyses of different vaccines (5 µg polysaccharide per mouse; labeled with Cy7‐SE) at injection sites (*n* = 3 per group) A) and in dLNs (*n* = 3 per group) B) in supine and prone positions, respectively, at different time points. C,D) We collected dLNs with NP‐OPS_KpO2_ (5 µg polysaccharide per mouse) on the third day postinjection, and then performed Flow cytometry (FCM) analysis C) and IF staining D). C) Proportion of CD4^+^ T cells among total dLN cells (*n* = 5 per group). D) Representative partial multicolor IF‐stained images of lymph node tissues (green: CD3^+^ T cells, red: CD4^+^ T cells, blue: nuclei). E,F) We collected dLNs with NP‐OPS_KpO2_ (5 µg polysaccharide per mouse) on the 5th day postinjection, and then performed FCM analysis E) and IF staining F). E) Proportion of Tfh cells (CXCR5^+^ and PD‐1^+^ among the CD4^+^ cell population) and B cells in GCs (GL7^+^ and CD95^+^ among the B220^+^ cell population) (*n* = 5 per group). F) Representative multicolor IF‐stained images of lymph node tissues (red: GC B cells, blue: nuclei). Data are presented as means ± SD. Each group was compared with NP‐OPS_KpO2_ using one‐way analysis of variance (ANOVA) and with Dunnett's multiple‐comparison test: ****P < 0.0001, ***P < 0.001, and *P < 0.05.

Because the duration of antigen in vivo and the accumulation of antigen in lymph nodes are related to immune response to vaccines, we detected the performance of the nanoconjugate vaccine that was purified as described above. We used sulfo‐cyanine7 succinimidyl ester (Cy7‐SE) fluorescent dye to covalently label three vaccine formulations: NP‐OPS_KpO2_, C‐OPS_KpO2_, and OPS_KpO2_. Then, each of these vaccines was subcutaneously injected into the tail base of BALB/c mice, fluorescent signals at the injection sites and in draining lymph nodes (dLNs) were detected at different time points. The signal intensity of OPS_KpO2_ decayed quickly at the injection site. Notably, we found NP‐OPS_KpO2_ to have better retention capability than C‐OPS_KpO2_ or OPS_KpO2_ (**Figure** **2**A). Meanwhile, the signal in dLNs was significantly enhanced in NP‐OPS_KpO2_‐treated mice, indicating a dramatic increase in lymph node accumulation (Figure 2B). It bears emphasizing that total‐accumulation intensity analysis of NP‐OPS_KpO2_ in dLNs revealed a greater than eightfold increase over OPS_KpO2_.

After confirming the high efficiency of lymph node drainage of NP‐OPS_KpO2_, we further analyzed the proportions of CD4^+^ T cells from dLNs on the 3rd day postinjection and observed a markedly significant increase in NP‐OPS_KpO2_‐treated mice (Figure 2C). This result was also confirmed by IF of dLNs, which showed more CD3^+^ CD4^+^ cells in tissue sections (Figure 2D; and Figure S8, Supporting Information). Moreover, the analysis of dLN on the 7th day postinjection revealed that the proportion of both T follicular helper (Tfh) cells and B cells in germinal centers (GCs) in the NP‐OPS_KpO2_ group were significantly higher than that in the other groups according to statistical analysis (Figure 2E). In addition, high levels of the proliferative marker Ki‐67 accumulated in the GC areas of dLNs from NP‐OPS_KpO2_‐treated mice, indicating that more B cells were activated in these mice (Figure 2F). These results indicated that the vaccine candidate NP‐OPS_KpO2_ excelled at stimulating humoral immune response and was apparently suitable in prophylactic‐vaccination applications.

### 2.3 Safety Evaluation of the Nanoconjugate Vaccine

**Figure 3:**
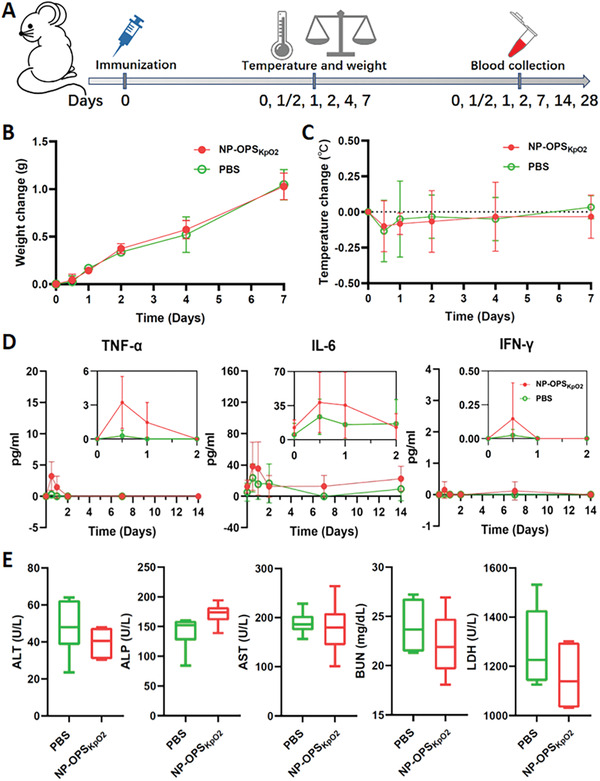
Safety estimation of nanoconjugate vaccine (*n* = 6 per group). A) Treatment schedule for the safety estimation experiment. B,C) Temperature B) and weight C) changes in mice immunized with NP‐OPS_KpO2_ (25 µg polysaccharide per mouse) 7 days postinjection. D) Cytokine levels (TNF‐*α*, IL‐6, IFN‐*γ*) in blood at different time points postimmunization. E) Detection of serum biochemical indices, including alanine aminotransferase (ALT), alanine aminotransferase (ALP), aminotransferase (AST), blood urea nitrogen (BUN), and lactate dehydrogenase (LDH), on the 28th day postimmunization.

Having confirmed the nanoconjugate vaccine's highly efficient immunostimulatory ability, we further evaluated its safety. BALB/c mice were immunized subcutaneously with NP‐OPS_KpO2_ (25 µg polysaccharide per mouse, 10 times of normal immune dose) or an equal volume of phosphate‐buffered saline (PBS), and then a series of experiments were performed at different time points (**Figure** **3**A). Weight and body temperature monitoring data revealed no abnormal changes in nanoconjugate vaccine‐treated mice during the observation period (Figure 3B,C). Meanwhile, in order to evaluate inflammatory conditions after immunization, we detected cytokine concentrations of tumor necrosis factor‐alpha (TNF‐*α*), interleukin‐6 (IL‐6), and interferon‐gamma (IFN‐*γ*) in serum. Little difference was found between the treatment and PBS groups, indicating that the nanoconjugate vaccine had no obvious systemic toxicity (Figure 3D). Furthermore, we detected certain serum biochemical indices—alanine aminotransferase (ALT), alanine aminotransferase (ALP), aminotransferase (AST), blood urea nitrogen (BUN), and lactate dehydrogenase (LDH)—on the 28th day postimmunization. All indices were within normal range (Figure 3E). Therefore, the nanoconjugate vaccine's outstanding safety and biocompatibility levels encouraged us to further evaluate its protective effect.

### 2.4 Evaluation of Specific Antibodies after Immunization with the Nanoconjugate Vaccine

**Figure 4:**
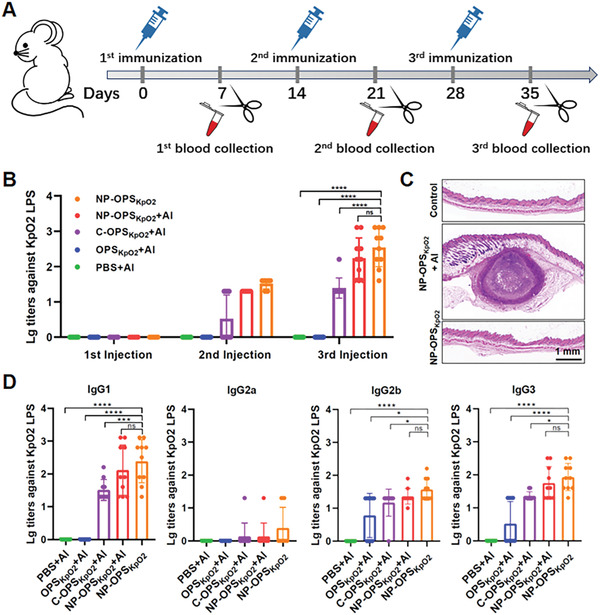
IgG responses of nanovaccine against *K. pneumoniae* serotype O2 LPS (*n* = 10 per group). A) Immunization schedule for titer evaluation. B) IgG titers against LPS of *K. pneumoniae* strain 355 were measured in the serum of BALB/c mice immunized with PBS + Al, OPS_KpO2_ + Al, C‐OPS_KpO2_ + Al, NP‐OPS_KpO2_ + Al, or NP‐OPS_KpO2_ 7 days postinjection. C) H&E staining of pathological sections of immune sites in mice immunized with NP‐OPS_KpO2_ or NP‐OPS_KpO2_ + Al. Control indicates normal mouse tissue. D) IgG subtype titers (IgG1, IgG2a, IgG2b, and IgG3) against LPS of *K. pneumoniae* strain 355 in immunized mice were measured after the third immunization. Data are represented as mean ± SD. Each group was compared using one‐way ANOVA with Dunnett's multiple‐comparison test: ****P < 0.0001, ***P < 0.001, *P < 0.05, and ns > 0.05.

We next used an animal evaluation model that we had previously established to assess humoral immune response.^[^ ^16a^ ^]^ BALB/c mice were immunized subcutaneously with one of five treatments—PBS + Al (having been approved in various human vaccines), OPS_KpO2_ + Al, C‐OPS_KpO2_ + Al, NP‐OPS_KpO2_ + Al, or NP‐OPS_KpO2_ (2.5 µg polysaccharide per mouse)—on days 0, 14, and 28. Blood was collected on days 7, 21, and 35 from tail veins to facilitate quantitation of antibodies against LPS of *K. pneumoniae* serotype O2 strain 355 (**Figure** **4**A; and Figure S9, Supporting Information), whose genome sequence has been uploaded to the EBI nucleotides database (https://www.ebi.ac.uk/ena/browser/view/GCA_903856825.1) and verified to be an O2 strain by Kaptive.^[^ ^17^ ^]^ Enzyme‐linked immunosorbent assay (ELISA)‐based measurement of IgG titers against *K. pneumoniae* strain 355 LPS in serum samples (Figure 4B) revealed increases after C‐OPS_KpO2_ + Al, NP‐OPS_KpO2_ + Al, and NP‐OPS_KpO2_ immunization, most obviously in the NP‐OPS_KpO2_‐treated mice. Notice that the addition of Al adjuvant did not help to increase the antibody titer caused by nanovaccine. Moreover, treatment with NP‐OPS_KpO2_ without Al adjuvant avoided the formation of subcutaneous granuloma, a side effect of that adjuvant (Figure 4C).

In addition, we measured titers of IgG antibody subtypes (IgG1, IgG2a, IgG2b, and IgG3) against *K. pneumoniae* strain 355 LPS in serum collected after the 3rd immunization (Figure 4D). ELISA results revealed that IgG1 was the main subtype in each group. The highest titer of each subtype appeared in the NP‐OPS_KpO2_‐immunized group, indicating that the nanoconjugate vaccine candidate (especially without Al adjuvant) could effectively stimulate humoral immune response and produce polysaccharide‐specific antibodies.

### 2.5 Prophylactic Effects in a Systemic Bacterial‐Infection Model

**Figure 5:**
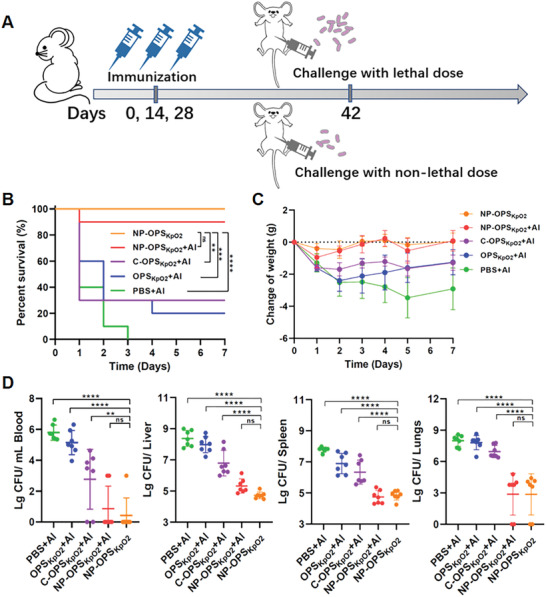
Evaluation of robust prophylactic effects against systemic bacterial infection. A) Treatment schedule for evaluation of systemic infection. B) Immunized mice (*n* = 10 per group) were injected i.p. with *K. pneumoniae* strain 355 (5 × 10^7^ CFU per mouse) 14 days after final immunization, and their survival times were monitored. C,D) Mice were challenged with *K. pneumoniae* strain 355 (2 × 10^7^ CFU per mouse). We determined changes in weight (*n* = 6 per group) and counted bacterial loads in blood and some organs (liver, spleen, and lungs) 20 h postchallenge (*n* = 7 per group). Data are presented as means ± SD. Survival analysis was calculated by log‐rank test: ****P < 0.0001, ***P < 0.001, and **P < 0.01. Each group in bacterial‐load analysis was compared using one‐way ANOVA with Dunnett's multiple comparison test: ****P < 0.0001, **P < 0.01, and ns > 0.05.

As the nanoconjugate vaccine candidate had successfully elicited LPS‐specific IgG antibodies, we further evaluated its protective efficacy by intraperitoneal (i.p.) challenge with different doses of the opportunistic MDR pathogen *K. pneumoniae* serotype O2 strain 355 (Figure S10, Supporting Information) on day 42 (14th day after the 3rd immunization; **Figure** **5**A). Initially, after immunization with one of five treatments (PBS + Al, OPS_KpO2_ + Al, C‐OPS_KpO2_ + Al, NP‐OPS_KpO2_ + Al, or NP‐OPS_KpO2_) as described above, mice were abdominally injected with a dose of 5 × 10^7^ CFU per mouse, and survival times were monitored for 7 days (Figure 5B). All PBS + Al‐vaccinated mice died rapidly, within 3 days. Survival rates in the OPS_KpO2_ + Al and C‐OPS_KpO2_ + Al groups had small increases, not exceeding 30%. By contrast, groups treated with NP‐OPS_KpO2_s demonstrated outstanding vaccine protection and had high survival rates. The survival rate of the group treated with NP‐OPS_KpO2_ without Al adjuvant even reached 100%. We also confirmed that this protective effect was due to the specific immune response from bacterial polysaccharides rather than from the NPs themselves (Figure S11, Supporting Information). These results demonstrated that the NP‐OPS_KpO2_ nanoconjugate vaccine candidate could provide sufficient protection against lethal challenge with *Klebsiella*.

To support the strong antipathogenic effects of immunization with our nanoconjugate vaccine against a high i.p. dose of *K. pneumoniae*, we also investigated its prophylactic effect against systemic infection with a lower dose of 2 × 10^7^ CFU per mouse after three immunizations, using the same methods described above. The weight of each challenged mouse was monitored for 7 days. Results showed that the weight of PBS + Al‐treated mice decreased continuously, and average loss was nearly 17% (about 3.5 g) by the 5th day (Figure 5C). Both OPS_KpO2_ + Al‐ and C‐OPS_KpO2_ + Al‐treated mice fared slightly better, losing about 12% (2.4 g) and 9% (1.7 g), respectively, by the 2nd day after challenge. NP‐OPS_KpO2_‐treated mice fared the best, seeming to lose little weight, whether with or without adjuvant. Bacterial‐clearance analysis at 20 h postchallenge in blood and organs (liver, spleen, and lungs) clearly revealed that bacterial loads in all such testing samples had greatly decreased in NP‐OPS_KpO2_‐immunized mice, and the greatest reduction was 99.99% in the blood, compared with the PBS + Al group (Figure 5D). Taken together, these results demonstrated that NP‐OPS_KpO2_ significantly outperformed C‐OPS_KpO2_ and OPS_KpO2_ in eliciting protection against severe systemic infection from *Klebsiella*.

### 2.6 Immune Effects of the Nanoconjugate Vaccine in a Pulmonary‐Infection Model

**Figure 6:**
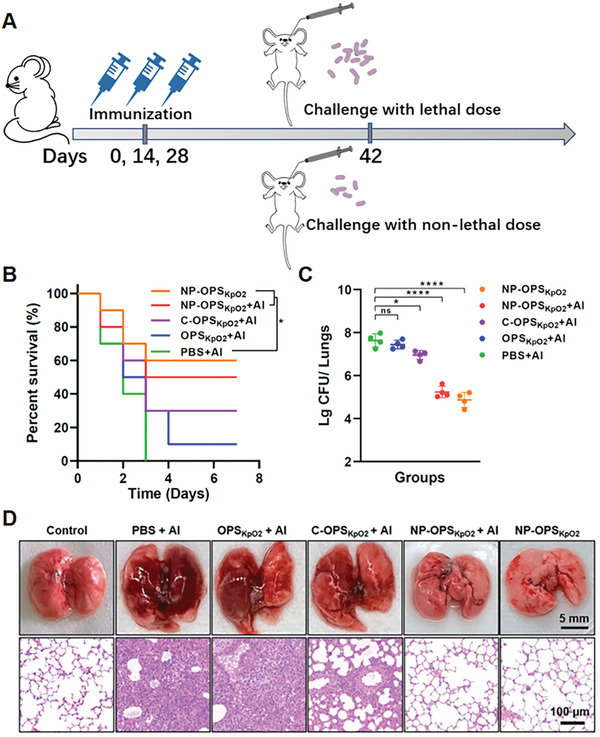
Strong protective efficacy in lungs against bacterial pulmonary infection. A) Treatment schedule for evaluation of pulmonary infection. B) Survival times of mice were monitored after challenge with *K. pneumoniae* strain 355 (2 × 10^8^ CFU per mouse) via intratracheal or endobronchial instillation 14 days after final immunization (*n* = 10 per group). C,D) Mice were challenged with *K. pneumoniae* strain 355 (1 × 10^8^ CFU per mouse). We counted bacterial loads in lungs (*n* = 4 per group) C) and observed the appearance of pathological sections of lungs D) at 40 h postchallenge. Data are presented as means ± SD. Each group in bacterial‐load analysis was compared using one‐way ANOVA with Dunnett's multiple‐comparison test: ****P < 0.0001, *P < 0.05, and ns > 0.05.

Considering that the lungs are the target organs of *K. pneumoniae*, we further established a pulmonary‐infection model to evaluate the protective effect of our nanoconjugate vaccine. Mice were challenged with different doses of *K. pneumoniae* strain 355 via intratracheal instillation by “Micro Sprayer” (Figure S12, Supporting Information).^[^ ^18^ ^]^ On the 14th day after the 3rd immunization with one of five treatments (PBS + Al, OPS_KpO2_ + Al, C‐OPS_KpO2_ + Al, NP‐OPS_KpO2_ + Al, or NP‐OPS_KpO2_) as described above (**Figure** **6**A), mice were challenged with a dose of 2 × 10^8^ CFU. All mice in the PBS + Al and OPS_KpO2_ + Al groups died quickly, within 4 days (Figure 6B). By contrast, the survival rate increased to 30% in the C‐OPS_KpO2_ + Al group. Notably, we observed further dramatic increases in protective effect in NP‐OPS_KpO2_‐immunized mice, reaching 50% and 60%, respectively, in the NP‐OPS_KpO2_ + Al and NP‐OPS_KpO2_ groups.

To further evaluate the vaccine's bacterial‐clearance capability in lungs after immunization, mice were challenged with a reduced dose of about 1 × 10^8^ CFU per mouse, and bacterial loads in lungs were measured about 40 h postinfection. Compared with the PBS + Al group, bacterial loads in OPS_KpO2_ + Al‐treated mice hardly decreased, but in C‐OPS_KpO2_ + Al‐treated mice they decreased by 41.4%. Although these decreases were substantial compared with those in C‐OPS_KpO2_ + Al‐treated mice, we measured even greater dramatic decreases in bacterial loads in NP‐OPS_KpO2_‐treated mice, especially those immunized without adjuvant: their bacterial loads decreased by >99% compared with PBS + Al‐, OPS_KpO2_ + Al‐, or C‐OPS_KpO2_ + Al‐treated mice (Figure 6C). We sacrificed some of the challenged mice at 40 h postinfection. In line with our observations of survival rates and bacterial loads, appearances and Hematoxylin and Eosin (H&E) staining analysis of dissected lungs revealed similar trends for the five treatments in injury and pathology. Of note, large areas of congestion and edema appeared in the PBS + Al and OPS_KpO2_ + Al groups, and, correspondingly, we observed severe pulmonary consolidation with alveolar exudate in tissue sections. Meanwhile, the lungs of nanoconjugate vaccine‐immunized mice greatly resembled those of the normal group, revealing the strong protective effect of the nanoconjugate vaccine on the target organs (Figure 6D). These results clearly suggested that the nanoconjugate vaccine could provide excellent prophylactic effects against *K. pneumoniae*.

## 3 Discussion

*K. pneumoniae* is a severe hospital‐acquired pathogen that causes high morbidity and mortality, especially in intensive care units (ICUs) and pediatric wards.^[^ ^19^ ^]^ It has recently attained notoriety as an infectious agent due to a growing number of infections and a lack of treatments. Of additional concern is the emergence of *K. pneumoniae* strains that have acquired additional genetic traits and become resistant to multiple antibiotics. In this study, we constructed a general nanoscale conjugate vaccine preparation system, and prepared a safe and efficient nanoconjugate vaccine against *K. pneumoniae*. Subsequent animal experiments demonstrated that our nanoconjugate vaccine could effectively stimulate humoral immune response and produce polysaccharide‐specific antibodies, thereby providing excellent protection against *K. pneumoniae* infection in different models.

This nanoconjugate vaccine was created using biosynthetic methods and produced in a modified laboratory *E. coli* strain. We found that by knockout of the unnecessary OPS synthesisi realted genes, such as *wbbH*‐*wbbL*, glycosylation efficiency could be obviously increased over sevenfold, and total sugar yield increased about twelvefold in a shake flask culture. We believe that culturing via fermenter will produce a higher total sugar yield. Moreover, this heterologous production system can be further engineered and utilized. For example, we will construct a versatile and efficient expression host through precise modification of the chassis cell and modularization of expression elements using synthetic biology technology. This modification, we believe, will not be the last.

In contrast to CPS antigens, the diversity of OPS structures in the LPSs of *Klebsiella* is limited. This means that the use of O‐antigen to prepare vaccines can provide a wide range of protective effects. Regrettably, the simple structure of O2‐polysaccharide results in lower immunogenicity, meaning that it is usually difficult to elicit effective immune protection with O2‐serotype OPS. Although we have shown that CTB‐based conjugate vaccines can induce almost 100% protection against various infections, such as *Shigella*, *S. paratyphi* A, and *B. abortus*,^[^ ^16^ ^]^ this kind of conjugate vaccine does not induce sufficient protection against *K. pneumoniae*. Therefore, we introduced our Nano‐B5 self‐assembly platform in this study. Our nanoconjugate vaccine was about 25 nm in size, within the optimal range of 15–100 nm for direct delivery to dLNs;^[^ ^20^ ^]^ indeed, it showed good lymph node targeting ability and ensured effective antigen accumulation in dLNs. And the nanoconjugate vaccine was highly capable of eliciting polysaccharide antigen‐specific immune responses. That is, we demonstrated that OPS with low immunogenicity could be changed into an effective antigen in our Nano‐B5 platform, indicating that polysaccharide antigen, with low immunogenicity, or conventional vaccine that cannot stimulate effective immune response could be applicable to this strategy in the future.

Besides, it bears mentioning that various modifications can be carried out through genetic manipulation to expand the application of our nanoconjugate vaccine preparation system. For instance, vaccines of *K. pneumoniae* serotype O1 could be further prepared by introducing the two additional genes *wbbY* and *wbbZ*. Meanwhile, the usage of our system could be extended to other bacterium with high level of biosafety, particularly those need to be cultured under strict and special conditions. The conjugate vaccines against other pathogens could be prepared by simply replacing the original polysaccharide gene clusters in this system with those of target bacteria. Moreover, a series of optimizations on the protein particles could be adopted to upgrade the nanoscale conjugate vaccine. For example, some functional components such as major histocompatibility complex (MHC) binding or dendritic‐cell (DC)‐targeting peptides could be fused into (or modified on) the NP to further improve specificity and vaccine performance.

Finally, the carrier protein, such as CTB used in this study, could elicit a high titer of antibodies (Figure S13, Supporting Information). Although the high immunogenicity of the carrier protein can effectively activate the immune response, using the same carrier for different glycoconjugate vaccines may result in the immune response dampening the effect of subsequent vaccination.^[^ ^21^ ^]^ This carrier induced epitope suppression (CIES) has been a great concern with proteinaceous carrier vaccines, such as conjugate vaccines and VLP vaccines.^[^ ^22^ ^]^ In addition to the reports that CIES could be overcome by high coupling densities, repeated injections, and/or higher doses of conjugate vaccine,^[^ ^22b^ ^]^ the design of our Nano‐B5 nanovaccines outperforms conventional vaccine technology especially in the term of their versatility, which enables extensive combinations of modular parts and antigen cargos for easily generating a potentially enormous diversity of nanovaccine structures. For example, we have designed and prepared three different nanocarriers (including CTBtri, LTBtri, and StxBtri) based on our Nano‐B5 platform.^[^ ^15^ ^]^ These B5 subunits (CTB, LTB, and StxB) of bacterial AB5 toxins have relative low sequence identity with each other, and all of them could self‐assembled into pentamers.^[^ ^23^ ^]^ Thus, we would expect that the B5 nanoparticle platform with diverse carrier proteins might be used as a general vaccination platform to carry different antigens in the future.

Overall, our results showed that OPS of *K. pneumoniae*, which is composed of galactose repeats, could be used as an effective antigen in nanovaccine preparation platform to evoke an effective immune response against infection. These findings will overcome the belief that polysaccharides with simple structures like *K. pneumoniae* are not suitable for vaccine design and might return many haptens to the list of candidate antigens for vaccines. To our knowledge, this was the first application of Nano‐B5 self‐assembly platform for preparation of a nanoconjugate vaccine in a general *E. coli* host system. Our approach thus offers an attractive platform technology for loading diverse polysaccharide antigen cargos to generate various high‐performance nanoconjugate vaccines.

## 4 Experimental Section

### Animals

All murine immunizations complied with ethical regulations for animal testing and research. All experiments were approved by and conducted in accordance with the guidelines of the Academy of Military Medical Sciences Institutional Animal Care and Use Committee (Ethics Approval Code IACUC‐DWZX‐2020‐042). Specific‐pathogen‐free (SPF) female BALB/c mice were purchased from Vital River Laboratory Animal Technology (Beijing, China) and experiments were conducted at the center.

### Bacterial Strains, Plasmids, and Growth Conditions

The bacterial strains and plasmids used in this study are listed in Table S1 (Supporting Information). All bacterial strains were cultured in Luria–Bertani (LB) broth or on solid LB medium containing 1.5% agar.

For recombinase expression, cells were first cultured in LB (with 50 µg mL^−1^ ampicillin) at 30 °C. 1 h before optical density at 600 nm (OD_600_) reached ≈0.5, L‐arabinose (final concentration at 5 mmol) was added, and the mixture was cultured at 30 °C for 1 h to induce recombinase expression . To eliminate temperature‐sensitive plasmids, cells were first cultured in LB (without antibiotic) at 37 °C and then transferred to new medium for subculture at 42 °C, and the subculture 2–3 times were repeated. For protein expression, cells were first cultured in LB at 37 °C to an OD_600_ of ≈0.6–0.8. Then, Isopropyl‐*β*‐D‐1‐thiogalactopyranoside (IPTG) (final concentration at 1 mmol) was added to induce protein expression at 30 °C for 10–12 h. When necessary, kanamycin and chloramphenicol (both 50 µg mL^–1^) were added to the culture medium.

### Construction of Gene Mutant Strains of *E. Coli*

The *waaL* knockout strain using the *λ*‐Red recombination system was constructed. First, the targeting fragment, a kanamycin‐resistant fragment flanked by FRT sites and homologous sequences of W3110, via polymerase chain reaction (PCR) using template pET‐*kan* were amplified. Here, the primer KO‐*waaL*‐F/R containing a 42 bp upstream or downstream sequence corresponding to the regions immediately adjacent to *waaL* was used. After purification, the products were introduced into W3110/pKD46‐competent cells by electroporation, and the transformed cells were cultured overnight at 30 °C. Then, colonies with the correct construct via PCR using primers *waaL*‐in‐F/R and *waaL*‐out‐F/R were identified. Finally, plasmid pCP20 was introduced to excise the antibiotic gene.

The *waaL*/*wbbH‐L* double‐knockout strain was constructed by a similar process based on strain W3110△*waaL*. Primer KO‐*wbbHL*‐F/R was used to amplify the targeting fragment, and primers *wbbHL‐*in‐F/R and *wbbHL‐*out‐F/R were used to identify the correct colonies. All primers mentioned above are listed in Table S2 (Supporting Information).

### LPS and OPS Preparation

LPS extracted by hot‐phenol method, details as follow. The culture was collected, washed, with PBS and then suspended in ddH_2_O. After freezing and thawing 3 times, an equal volume of 90% phenol was added and followed by vigorous shaking at 68 ℃ for 15 min. Then centrifugation was performed. The water layer was collected in a new tube and the phenol layer was re‐extracted with ddH_2_O, repeated twice. The collected water layer was dialyzed into ddH_2_O for at least 3 days, then DNase (5 µg mL^−1^; Solarbio, Beijing, China), RNase (5 µg mL^−1^; Solarbio), and proteinase K (20 µg mL^−1^; Solarbio) were sequentially added and incubated at the optimal temperature of each enzyme. After a boiling water bath for 10 min, the supernatant was collected as the LPS solution. Glacial acetic acid was added to the extracted LPS solution with a final concentration of 1% (v v^−1^). After boiling water bath for 90 min, the mixture was adjusted pH to 7.0 with 1 × 10^−3^ m NaOH and centrifuged at 40 000 × g for 5 h, the supernatant was collected as the OPS solution.

### Silver Staining

LPS samples extracted by the hot phenol method were mixed with equal volumes of 2 × SDS loading buffer (100 × 10^−3^ m Tris‐HCl [pH 6.8], 3.2% w v^−1^ SDS, 0.04% w v^−1^ bromophenol blue, 16% v v^−1^ glycerol, and 40 × 10^−3^ m DL‐dithiothreitol) and bathed in boiling water for 10 min. Then, an aliquot of each sample (10 µL) was separated on SDS‐PAGE. Next, this gel was first placed into fixing solution (40% ethanol, 5% acetic acid, double‐distilled water [ddH_2_O] added to 100 mL) for 30 min. Then, the fixing solution was replaced with sensitizing solution (7 g sodium acetate, 0.2 g sodium thiosulfate, 30 mL ethanol, 0.25 g glutaraldehyde, ddH_2_O added to 100 mL) for 30 min and the gel was washed 3 times, 15 min each time, in copious ddH_2_O. After that, the water was drained off, poured in freshly prepared staining reagent (2.5 g silver nitrate, 40 µL formaldehyde, ddH_2_O added to 100 mL) and agitated the mixture vigorously for 20 min. Finally, after two 1 min washes in ddH_2_O, the gel was placed into developer solution (0.75 g sodium carbonate, 3 µL 5% sodium thiosulfate, 40 µL formaldehyde, ddH_2_O added to 100 mL) for 2–5 min with gentle agitation. Development was terminated when developer solution was replaced with stop solution (1.46 g disodium ethylenediamine tetraacetic acid [EDTA], ddH_2_O added to 100 mL).

### Coomassie Blue Staining and Western Blotting

After IPTG induction, cells were pelleted, washed, suspended in PBS and mixed with an equal volume of 2 × SDS‐loading buffer. After a boiling‐water bath for 10 min, an aliquot of each sample (10 µL) was separated on SDS‐PAGE, after which samples were stained with Coomassie Blue and transferred them to nitrocellulose membranes. The nitrocellulose membranes were blocked with blocking buffer (wash buffer consisting of Tris‐NaCl buffer + Tween‐20 [TBST], with 5% skim milk powder), incubated with primary antibodies for 30 min, washed three times in wash buffer, incubated with secondary antibodies for 30 min, washed more three times in wash buffer and visualized on an Imaging System Tanon 5200 (Tanon Science & Technology Co., Ltd., Shanghai, China) after added color developing solution (Thermo Fisher Scientific, Waltham, USA). Horseradish peroxidase (HRP)‐conjugated anti‐6 × His Tag antibodies (Abmart Shanghai Co., Ltd., Shanghai, China) were used to detect proteins with His tags and antibodies against *K. pneumoniae* serotype O2 to detect the glycan of glycoproteins. This antibodies against *K. pneumoniae* serotype O2 by immunizing Japanese white rabbits with *K. pneumoniae* strain 355 whole bacteria and blocking with *E. coli* W3110 cell lysates were produced. HRP‐conjugated anti‐rabbit IgG (Transgen Biotech, Inc., Beijing, China) was used as the secondary antibody.

### Purification of Glycosylated Protein

After expression, cells were collected and suspended in buffer A (20 × 10^−3^ m Tris‐HCl, pH 7.5, 10 × 10^−3^ m imidazole, and 0.5 m NaCl), 10 mL per 1 g cells. After disruption by a high‐pressure homogenizer (Ph.D. Technology LLC, Saint Paul, USA), the supernatant of the lysate onto a pre‐equilibrated nickel affinity column with cOmplete His‐Tag Purification Resin (Roche, Penzberg, Germany) at a flow rate of 2 mL per min was loaded. Then, this column was washed with about 10 column volumes of buffer A and 10 column volumes of buffer B (20 × 10^−3^ m Tris‐HCl, pH 7.5, 25 × 10^−3^ m imidazole and 0.5 m NaCl), and the target glycoprotein was harvested via elution with 100% buffer C (20 × 10−3 m Tris‐HCl, pH 7.5, 0.5 m imidazole and 0.5 m NaCl). The eluent to almost 5 mL using a 10‐kDa‐cutoff centrifugal filter (Merck, Darmstadt, Germany) was concentrated. Finally, the concentrated sample was flowed through column containing 200 mL Superdex‐200 (GE Healthcare, Marlborough, USA) in a mobile phase of PBS at a flow rate of 1 mL min^−1^. All purified extracts were collected into 2 mL tubes and analyzed them on SDS‐PAGE.

### PAS Staining of Polysaccharide

Purified extracted samples were mixed with equal volumes of 2 × SDS loading buffer and bathed in boiling water for 10 min, and then an aliquot of each sample (30 µL) was separated on SDS‐PAGE. Next, the gel was stained by a Pierce Glycoprotein Staining Kit (Thermo Fisher Scientific). First, the gel was immersed completely in 50% methanol, fixed for 30 min, and washed twice with 3% acetic acid, 10 min each time. Then, the gel was transferred to oxidizing solution for 15 min and washed the gel three times with 3% acetic acid, 5 min each time. After that, the oxidizing solution was replaced with glycoprotein staining buffer for 15 min and transferred the gel to reducing solution with gentle agitation. Last, the gel was washed extensively with 3% acetic acid and then with ddH_2_O.

### Lymph Node Imaging Assay

OPS_KpO2_ bound to a simple peptide, C‐OPS_KpO2_, and NP‐OPS_KpO2_ were tested. OPS_KpO2_ bound to a simple peptide was prepared as follows: C‐OPS_KpO2_ was digested by protease K (50 µg mL^−1^) at 58 °C overnight, and then the OPS bounding a simple peptide was achieved by a 10 kDa cutoff centrifugal filter (Merck) to remove small peptide segments. The three above mentioned vaccine candidates were labeled with Cy7‐SE Dye (Fanbo Biochemicals, Beijing, China) at 4 °C overnight, and unbound dyes were removed using the 10 kDa cutoff centrifugal filter (Merck) for four times. In order to ensure the normalization of the fluorescent labels in each group of mice, the fluorescence intensity of each sample was tested in vitro before injection to ensure the consistence when injected into the mice. Then, each sample was subcutaneously injected into the tail base of BALB/c mice, and the animals and their dLNs on an IVIS Spectrum In Vivo Imaging System (PerkinElmer, Waltham, USA) were imaged.

### FCM Detection of Immune Cell Differentiation

Vaccine candidates NP‐OPS_KpO2_, C‐OPS_KpO2_, and OPS_KpO2_ were each prepared at the same dose. All candidates and PBS control were subcutaneously injected into the tail base of BALB/c mice. Some of the treated mice were sacrificed on the 3rd day postinjection and analyzed the proportions of CD4^+^ and CD8^+^ T cells in their dLNs via FCM. Moreover, on the 7th day postinjection, the proportions of Tfh cells, and B cells in GCs were analyzed.

First, isolated popliteal dLNs to precooled PBS and triturated them into single‐cell suspensions. Then, cells were stained with different combinations of FCM antibodies, including APC‐conjugated anti‐mouse CD3 (eBioscience, San Diego, USA), FITC conjugated anti‐mouse CD4 (BioLegend, San Diego, USA), PE‐conjugated anti‐mouse CD8a (eBioscience), PE conjugated anti‐mouse PD‐1 (BioLegend), Brilliant Violet 421 conjugated anti‐mouse CXCR5 (BioLegend), APC‐conjugated anti‐mouse B220 (BioLegend), Pacific Blue conjugated anti‐mouse GL‐7 (BioLegend), and PE conjugated anti‐mouse CD95 (BioLegend) for 30 min at 4 °C. After washing them with staining buffer (eBioscience), the cells were dispersed in 500 µL PBS and analyzed them on a CytoFLEX Flow Cytometer (Beckman Coulter Life Sciences, Brea, USA).

### Safety Estimation

NP‐OPS_KpO2_ was injected subcutaneously. Body temperature and weight were measured on days 0, 0.5, 1, 2, 4, and 7; blood was collected on days 0, 0.5, 1, 2, 7, 14, and 28 from tail veins; and serum was separated after centrifugation. On days 0, 0.5, 1, 2, 7, and 14, cytokine concentrations were determined in serum using three commercially available ELISA kits: Mouse TNF‐*α*, IL‐6, and IFN‐*γ* Precoated ELISA Kits (all from Dakewe Medical Equipment Co., Ltd., Shenzhen, China). On day 28, serum levels of alanine aminotransferase (ALT), aspartate transaminase (AST), alkaline phosphatase (ALP), blood urea nitrogen (BUN), and lactate dehydrogenase (LDH) using a Chemray 240 automatic biochemical analyzer (Rayto Life and Analytical Sciences Co., Ltd., Shenzhen, China) were determined.

### Immunization Experiments

Five groups of 6‐week‐old female BALB/c mice with a vaccine formulation (100 µL per mouse) on days 0, 14, and 28 were subcutaneously injected. Groups consisted of NP‐OPS_KpO2_, NP‐OPS_KpO2_ + Al (General Chemical Corp., Brighton, USA; as 10% of final volume), C‐OPS_KpO2_ + Al, OPS_KpO2_ + Al, and PBS +Al . Blood was collected on the 7th day postinjection from tail veins. The bacteria used in the challenge were passaged in a volume of 1:100 with freshly cultured bacterial solution and cultured in LB liquid medium in shaker (220 r min^−1^) at 37 °C. When the OD_600_ reached 2.0 (about 10^9^ CFU mL^−1^), bacteria were diluted with normal saline as needed for challenge experiments. At the same time, in order to prevent undiscovered operating errors, bacterial dilution and drop plate counting during each operation to determine the actual challenge dose were again carried out. Intraperitoneal challenge with bacterium was performed on the 14th day after the 3rd immunization by an injector. Intratracheal instillation was also performed on the 14th day after the 3rd immunization by the Micro Sprayer (Huironghe, Beijing, China), details as follow. First, the tracheal opening of the anesthetized mouse was found out by laryngoscope, and then the sprayer was inserted into the trachea about 25 mm through the mouse's larynx, and 50 µL of the solution was atomized into the lungs through the plunger.

### ELISA

96‐well plates were coated with LPS_KpO2_ extracted from *K. pneumoniae* stain 355 (10 µg well^−1^), incubated them at 4 °C overnight, washed them three times with wash buffer (PBS with 0.05% Tween 20) and dried them. Plates were blocked with blocking buffer (wash buffer with 5% skim milk powder; 200 µL well^−1^) at 37 °C for 2 h. After drying the plates, they were incubated in serially diluted serum (100 µL well^−1^) from each immunized mouse at 37 °C for 1 h. Next, plates were washed another three times and dried. Diluted HRP‐linked goat‐anti‐mouse antibodies (IgG, IgG1, IgG2a, IgG2b, and IgG3 [Abcam, Cambridge, UK]) (100 µL well^−1^) were added, and plates were incubated for another 1 h at 37 °C. After the washing step, a Soluble TMB Kit (CWBio, Beijing, China) was used to initiate a color‐producing reaction and measured the absorbance of each well at an OD of 450 nm.

### Determination of Bacterial Loads in Blood and Organs

Blood (10 µL) was collected from each mouse into a pipette, quickly placed it into a 1.5 mL microcentrifuge tube with normal saline (990 µL) and then mixed it by inversion as the initial sample. Organs (liver, spleen, and lungs) were removed, homogenized with normal saline (1 mL) and then collected in 2.0 mL microcentrifuge tubes as initial samples. After placed for 15 min, the supernatant of each initial sample was diluted and cultured on solid LB medium. The bacterial colonies were counted after culturing overnight at 37 °C.

### H&E Staining

Tissue samples were removed and fixed with 4% paraformaldehyde (Solarbio, Beijing, China), and then embedded and sectioned. Next, conventional baking, dewaxing, and hydration were performed. Sections were stained using a H&E Staining Kit (Solarbio), then dehydrated, cleared, and mounted in synthetic resin.

### Statistical Analyses

Antibody titers and bacterial loads were log_10_‐transformed. All the data were expressed as means ± SD and all statistical analyses were conducted using GraphPad Prism software version 8.0 (GraphPad Software, Inc., San Diego, USA). Sample sizes (*n*) were mentioned on each figure captions. Data were analyzed via one‐way ANOVA with Dunnett's multiple comparison test for multiple‐group comparation. Log‐rank test was used in survival analysis. Differences were considered statistically significant at *P* < 0.05 (****P < 0.0001, ***P < 0.001, **P < 0.01, and *P< 0.05).

## Conflict of Interest

The authors declare no conflict of interest.

## Supporting information

Supporting Information

## Data Availability

The data that support the findings of this study are available from the corresponding author upon reasonable request.
