# Supplementary material for: Production of a Promising Biosynthetic Self‐Assembled Nanoconjugate Vaccine against Klebsiella Pneumoniae Serotype O2 in a General Escherichia Coli Host
Source: Adv Sci (Weinh). 2021 May 24;8(14):2100549. doi: 10.1002/advs.202100549 (PMC8292882; doi:10.1002/advs.202100549)
Supplement: Supplementary file 1 — Supporting Information [file ADVS-8-2100549-s001.pdf]

© 2021 Wiley-VCH GmbH

ADVANCED SCIENCE
Open Access

Supporting Information

for *Adv. Sci.*, DOI: 10.1002/advs.202100549

Production of a Promising Biosynthetic Self-assembled Nanoconjugate Vaccine against *Klebsiella pneumoniae* Serotype O2 in a General *Escherichia coli* Host

Zhehui Peng, Jun Wu, Kangfeng Wang, Xin Li, Peng Sun, Lulu Zhang, Jing Huang, Yan Liu, Xiaoting Hua, Yunsong Yu, Chao Pan, Hengliang Wang*, and Li Zhu**

Supporting Information

Production of a Promising Biosynthetic Self-assembled Nanoconjugate Vaccine against *Klebsiella pneumoniae* Serotype O2 in a General *Escherichia coli* Host

Zhehui Peng, Jun Wu, Kangfeng Wang, Xin Li, Peng Sun, Lulu Zhang, Jing Huang, Yan Liu, Xiaoting Hua, Yunsong Yu, Chao Pan, Hengliang Wang*, and Li Zhu**

Table S1. Bacterial strains and plasmids used in this study.

Bacterial Strains and Plasmids	Characteristic [a)]	Source
K. pneumoniae strain 355	Serotype O2, CRKP	Sir Run Run Shaw Hospital, Zhejiang University School of Medicine
E. coli W3110	Gene *wbbL* was inactivated	Laboratory stock
E. coli W3110△*waaL*	Without gene *waaL*	This work
E. coli W3110△*waaL*△*wbbH-L*	Without *waaL*, *wbbH*, *wbbI*, *wbbJ*, *wbbK*, and *wbbL* genes	This work
E. coli W3110/pACYC184-OPS_{KpO2}	Could express O-polysaccharide of *K. pneumoniae* O2a on the surface of the bacteria	This work
E. coli W3110△*waaL*/ pET28a-*pglL-CTB* pACYC184-OPS_{KpO2}	Could successfully express glycolprotein C-OPS_{KpO2}	This work
E. coli W3110△*waaL*△*wbbH-L*/ pET28a-*pglL-CTB* pACYC184-OPS_{KpO2}	Increased efficiency in expression of glycolprotein C-OPS_{KpO2}	This work
E. coli W3110△*waaL*△*wbbH-L*/ pET28a-*pglL-NP* pACYC184-OPS_{KpO2}	Could efficiently express self-assembled nanoscale glycolprotein NP-OPS_{KpO2}	This work
S.paratyphi A 50973△*waaL*/ pET28a-*pglL-NP*	Could successfully express glycolprotein NP-OPS_{SpA}	Laboratory stock
pKD46	Used for λ-red recombination, araC-ParaB, Ap^r	Laboratory stock
pET-Kan	Carries a kanamycin resistance gene flanked by FRT sites, Kan^r	Laboratory stock
pCP20	Used for the removal of kanamycin resistance gene, Cm^r	Laboratory stock
pET28a-*pglL-CTB*	Encoded PglL and 6 × His-tagged CTB and fused DsbA signal peptide at N-terminus and glycosylation sequence (4573) fragment at C-terminus, both of them under control of tac promoter, Kan^r	Laboratory stock
pET28a-*pglL-NP*	Encoded PglL and C-terminal trimer-forming peptide at N-terminus, 6 × His-tagged CTB and fused DsbA signal peptide in the middle and PilE4573 fragment at C terminus, both of them under control of tac promoter, Kan^r	Laboratory stock
pACYC184-OPS_{KpO2}	Encoded O2 O-polysaccharide of *K. pneumoniae*, Cm^r	This work

[a)] Abbreviations: araC, 1- β- D -arabinofuranosylcytosine; FRT, FLP recombination target.

Table S2. All primers used to construct and confirm gene mutants in this study.

Primer	Sequence
KO-*waaL*-F:	AACAGTCAAGCAGTTTTGGAAAAGTTATCATCATTATAAAGGGTGTAGGCTGGAGCTGC
KO-*waaL*-R:	GTTGTATAGATAAGAAGTGAGTTTTAACTCACTTCTTAAACTATGGGAATTAGCCATGG
waaL-in-F:	CTCCAATGCCCCTCTTAGCC
waaL-in-R:	GCGCTAACACAAACCAGAGC
waaL-out-F:	ACTCCGGAATATCCCTCGCA
waaL-out-R:	TTAACGGCGGCACTGGATAG
KO-*wbbHL*-F:	GCCGCTCTTTATCAAGTGAAAAATATAATGAGTACGGATTAAGTGTAGGCTGGAGCTGC
KO-*wbbHL*-R:	GTATAAATAGCTTATCCATGCTTATATGCTTACGGCTTTATAATGGGAATTAGCCATGG
wbbHL-in-F:	TCGAAACCTGTAGTGGTGCC
wbbHL-in-R:	GCCACGACCCTTTAACCTCA
wbbHL-out-F:	TGAGACGGAACGCCATGAAT
wbbHL-out-R:	TCTTCTCACGGGAACGGTTG

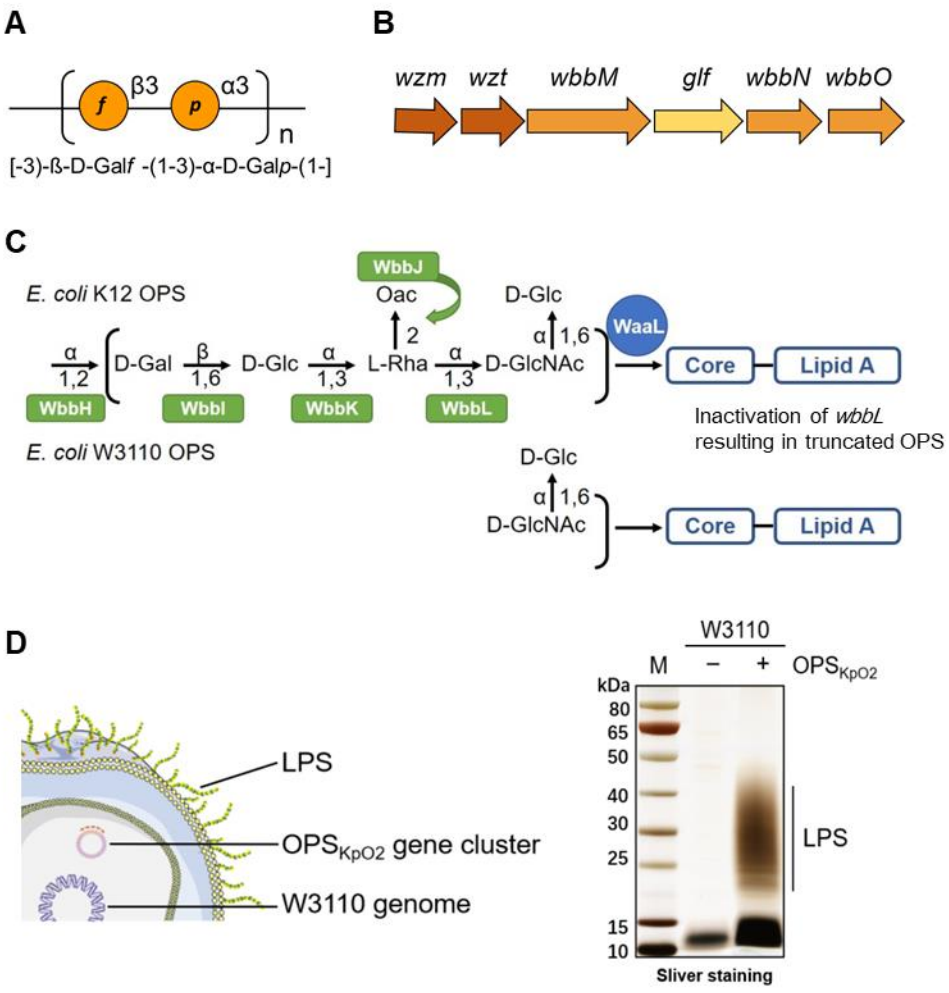

Figure S1. *K. pneumoniae* serotype O2 O-polysaccharide related informations and expresstion in *E. coli*.

The OPS of *K. pneumoniae* serotype O2 is composed of alternating α-D-Galp and β-D-Galf residues (A), which are encoded by a 7-kb gene cluster containing *wzm*, *wzt*, *wbbM*, *glf*, *wbbN*, and *wbbO* genes (B). This gene cluster together with its upstream promoter region were cloned into plasmid pACYC184 (named pACYC184-OPS_{KpO2}) and then transformed it into wild-type W3110, which lacked complete OPS structures due to inactivation of *wbbL* (C). After culturing, LPS was extracted from bacterium and silver staining was performed. Typical ladder-like bands of LPS were detected in strain W3110 containing pACYC184-OPS_{KpO2} (W3110/ pACYC184-OPS_{KpO2}) but not in the empty host strain (D).

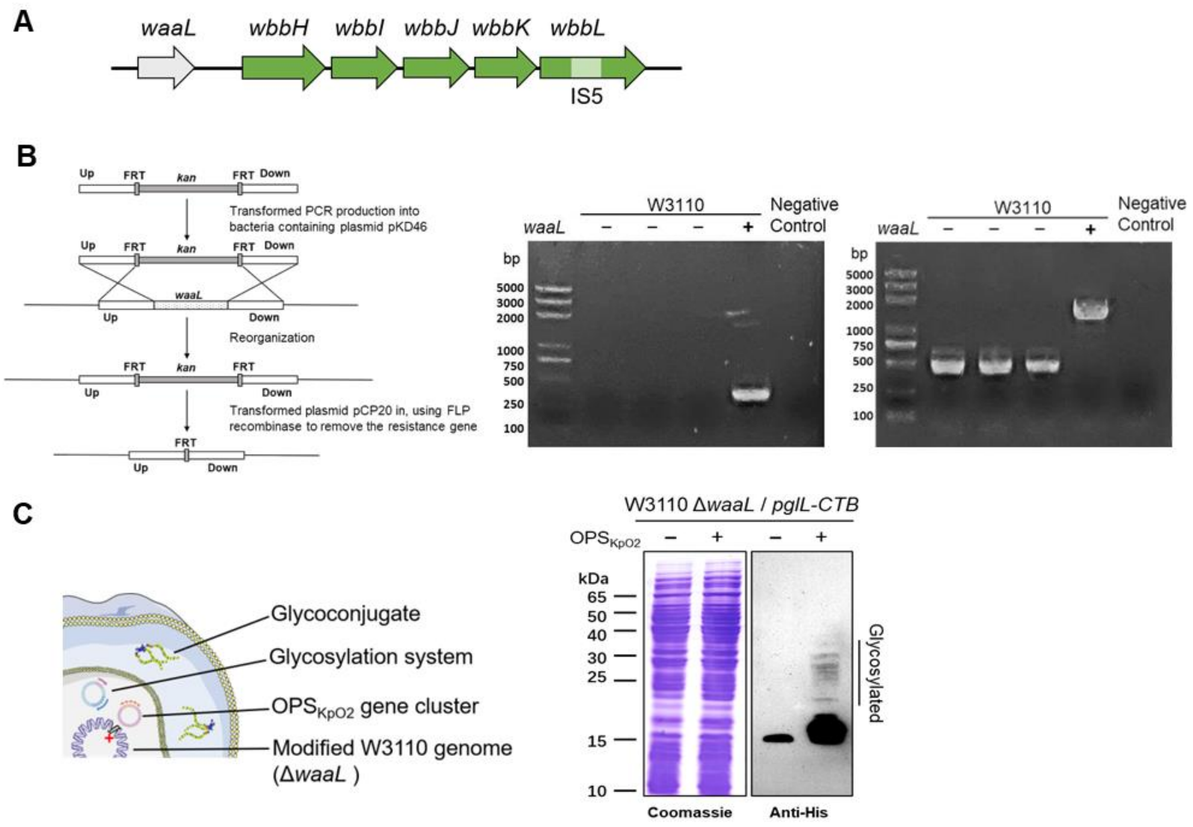

Figure S2. Establishment of glycosylation system in *E. coli.*

(A) Genes involved in W3110 O-polysaccharide synthesis. (B) Gene *waaL* was knocked out via λ-Red recombination system to avoid it competing with the next introduced PglL for UndPP-glycan substrate, and named this modified host W3110△*waaL*. Flowchart of *waaL* knockout (left) and PCR verification of mutant by inside and outside primers (right). (C) Western blot analysis of PglL-mediated O-linked CTB glycosylation with antibodies against 6 × His Tag in the whole-cell lysates of W3110△*waaL*/ pET28a-*pglL-CTB* pACYC184-OPS_{KpO2} showed typical ladder-like bands of C-OPS_{KpO2} in the range of 25-30 kDa.

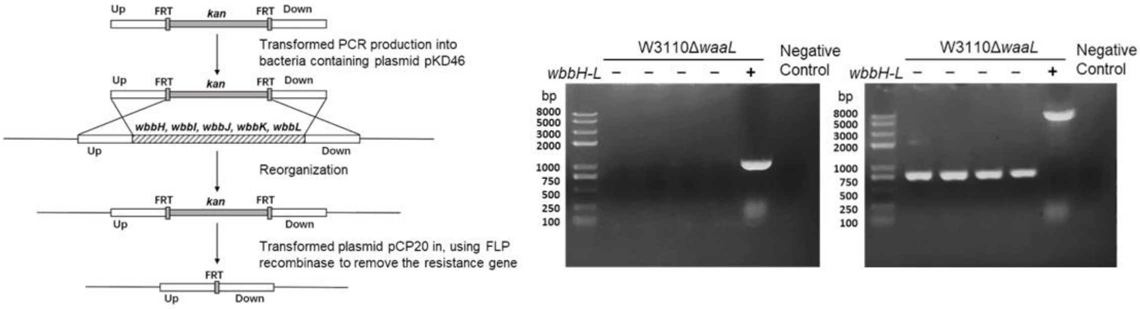

Figure S3. Gene fragment *wbbH-L* knockout flowchat and PCR verification.

Gene *wbbH-L* was knocked out via λ-Red recombination system to avoid the possible interference of the remaining OPS synthesis genes of *E.coli* host, and named this modified host W3110△*waaL*△*wbbH-L*. Flowchart of *wbbH-L* knockout (left) and PCR verification of mutant by inside and outside primers (right).

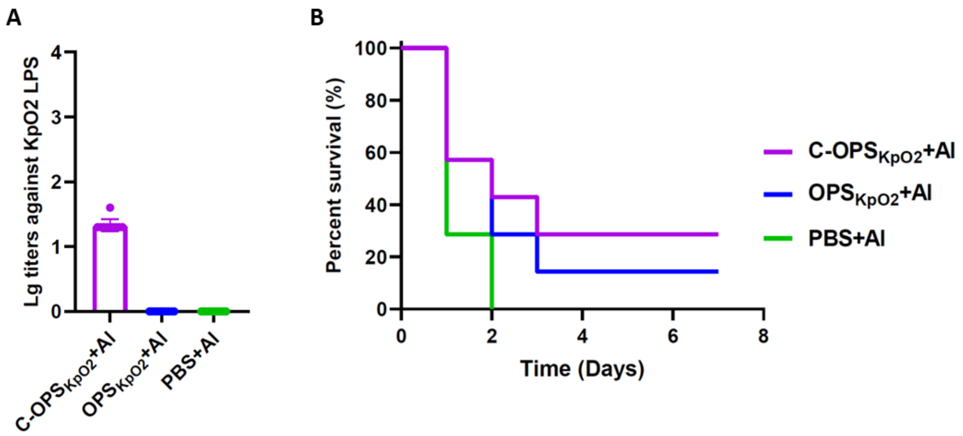

Figure S4. Detection of immune effects of C-OPS$_{KpO2}$.

BALB/c mice (6 weeks old; n = 7 per group) were immunized with one of three treatments: PBS + Al, OPS$_{KpO2}$ + Al, or C-OPS$_{KpO2}$ + Al (2.5 µg polysaccharide per mouse) on days 0, 14, and 28. Blood was collected on days 35 from tail veins to facilitate quantitation of antibodies against *K. pneumoniae* 355 LPS via ELISA (A). Immunized mice were injected i.p. with *K. pneumoniae* 355 (5×10^7 CFU per mouse) 14 days after final immunization, and their survival times were monitored (B).

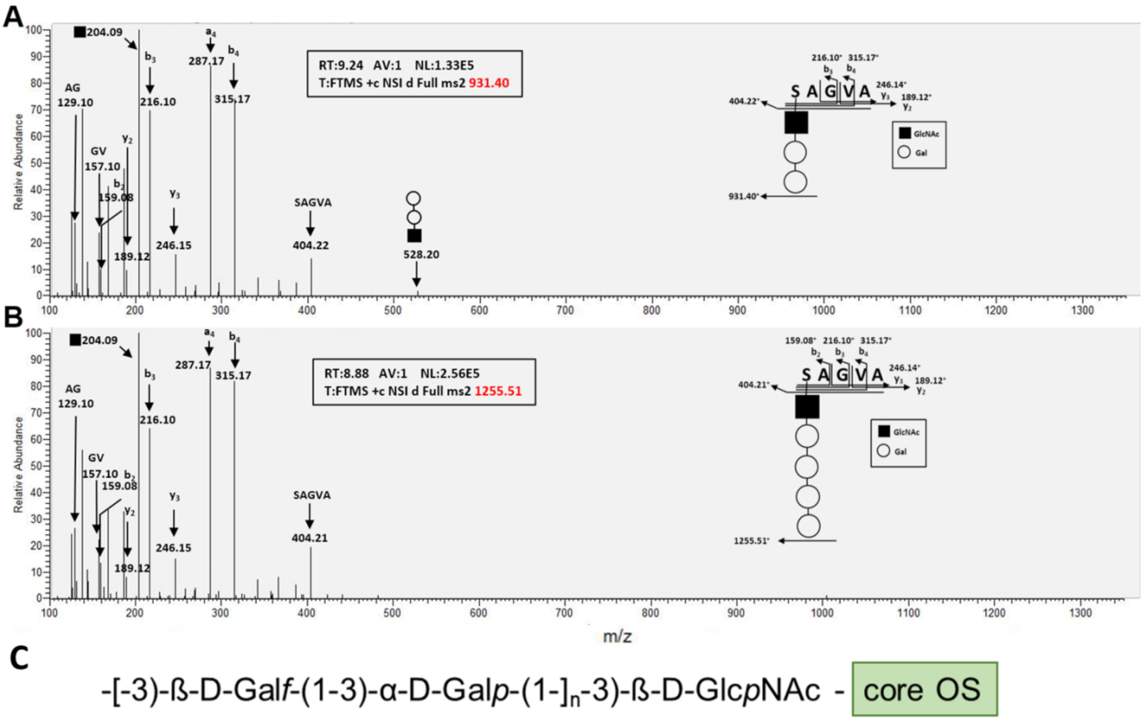

Figure S5. Identification of polysaccharide structure by LC-MS/MS.

The results revealed the expected-charged peptide SAGVA (m z^{-1} 404.21^{+}); the serine can be covalently bound to polysaccharides. Characterization of the mass-to-charge ratios of the breakdown products from collision induced dissociation verified the chemical structure of the sugar units. Since the O2 polysaccharide is a repetition of two galactoses on GlcNAc, two types of sugar chain structures (one repeat and two repeats) were identified from spectrums of singly charged ion at m z^{-1} 931.40^{+} (A) and 1255.51^{+} (B) respectively. Given that this targeted peptide only carry one positive charge, MS/MS spectra of singly charged ions released by Proteinase K digestion were collected in our mass spectrometry analysis. The mass-to-charge ratio are 931.40 or 1255.51 respectively when the peptide SAGVA coupled with one or two repeating units. These results revealed that the polysaccharide conjugated vaccine fabricated in *E. coli* bear the same OPS structure as the of *K. pneumoniae* serotype O2 strains (C).

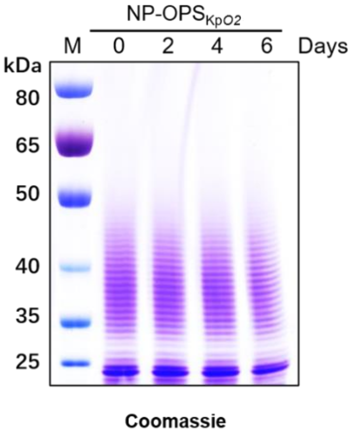

Figure S6. SDS-PAGE size stability analyses of NP-OPS$_{KpO2}$.

Samples were detected at different time points after filtering with an 0.22-μm filter and incubation at 37 ℃.

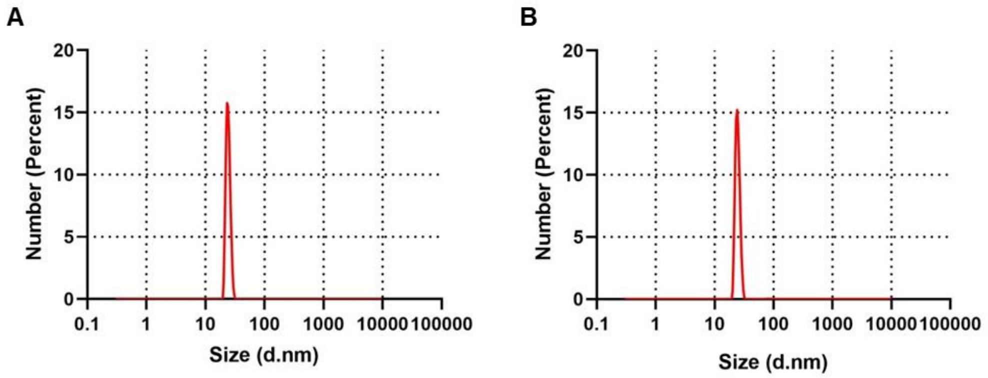

Figure S7. DLS size stability analyses of NP-OPS$_{\mathbf{KpO2}}$.

Analysis results before lyophilization (A) and after reconstitution (B).

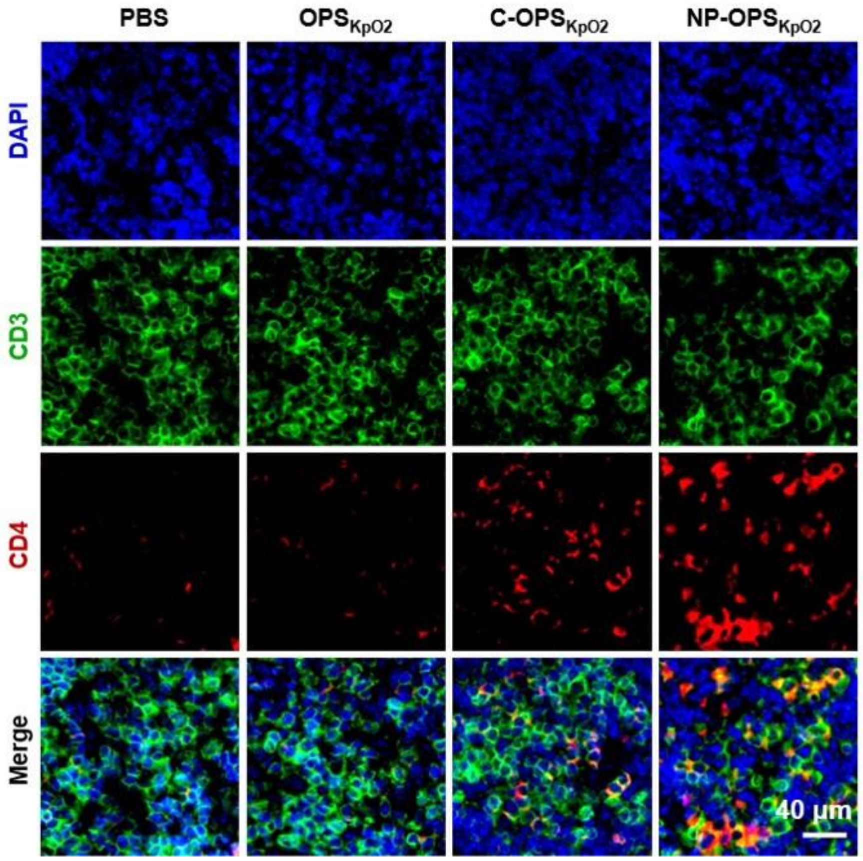

Figure S8. Multicolor images of IF-stained lymph node tissues.

Green: $CD3^{+}$ T cells, red: $CD4^{+}$ T cells, blue: nuclei.

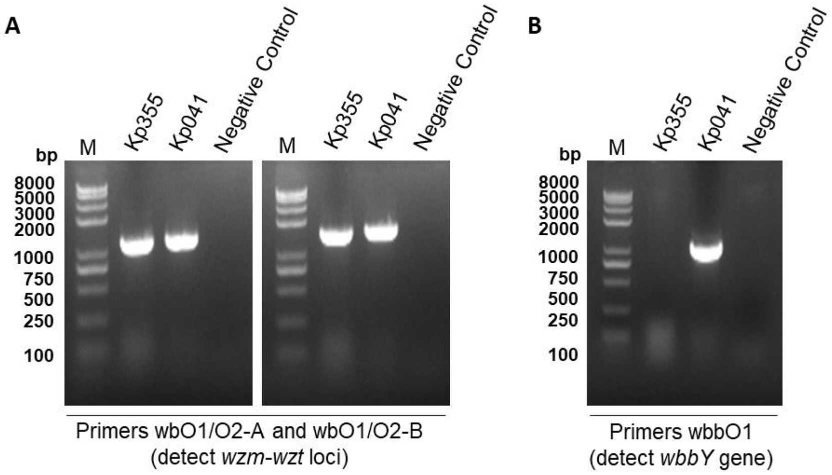

Figure S9. Identification of *K. pneumoniae* strain 355.

The primers wbO1/O2-A and primers wbO1/O2-B were used to amplify the consensus sequence, located at the *wzm-wzt* loci of the polysaccharide synthesis gene clusters, in strain Kp355 and Kp041 (an O1 serotype strain) (A). Following, primers wbbO1 were used to amplify the *wbbY* loci which belong to the O1 serotype (B). Therefore, the O2 serotype can be determined according to this method, due to the presence of the *wzm-wzt* loci and the lack of *wbbY* loci.

Antibiotic	MIC (mg/L)
Imipenem	32
Meropenem	128
Ertapenem	>128
Tigecycline	2
Colistin	0.06
Ceftazidime-avibactam	2
Fosfomycin	32
Aztreonam	>64
Amikacin	2

Figure S10. Antibiotic resistances of *K. pneumoniae* strain 355.

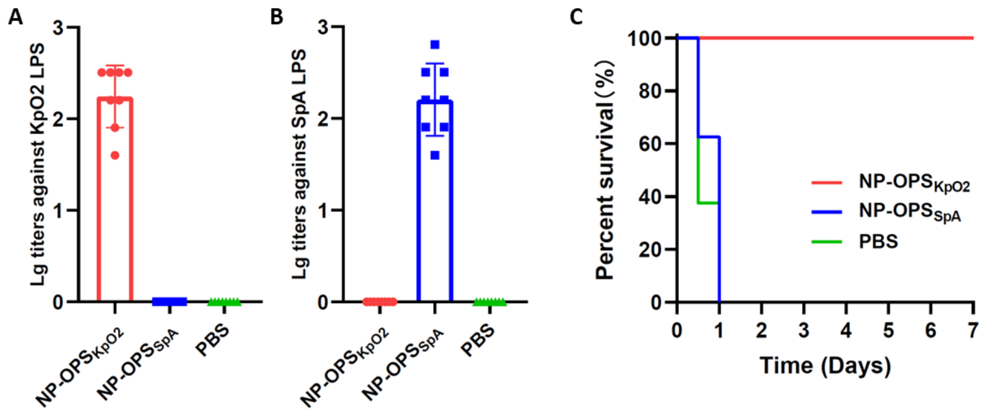

Figure S11. Detection of cross immune effects of NPs.

BALB/c mice (6 weeks old; *n* = 8 per group) were immunized with NP-OPS$_{KpO2}$ or NP-OPS$_{SpA}$ (NP connected with *S. paratyphi* A 50973 OPS; 2.5 μg polysaccharide per mouse) on days 0, 14, and 28. Blood was collected on day 35 from tail veins to facilitate quantitation of antibodies against *K. pneumoniae* 355 LPS (A) and *S. paratyphi* A 50973 LPS (B) via ELISA. Immunized mice were injected i.p. with *K. pneumoniae* 355 (5×10^7 CFU per mouse) 14 days after final immunization, and their survival times were monitored (C).

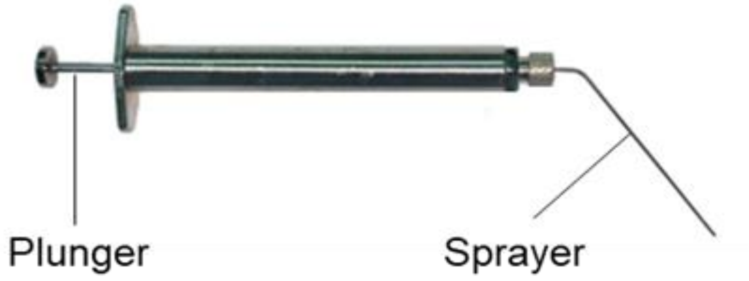

Figure S12. Micro Sprayer used to establishe the mouse lung infection model.

Firstly, the tracheal opening of the anesthetized mouse was found out by laryngoscope, and then the sprayer was inserted into the trachea about 25 mm through the mouse's larynx, and 50 μL of the solution was atomized into the lungs through the plunger.

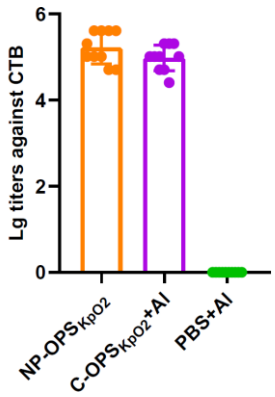

Figure S13. ELISA evaluation of IgG titers against CTB in serum samples.

IgG titers against CTB were measured in the serum of BALB/c mice (6 weeks old; *n* = 10 per group) immunized with PBS + Al, C-OPS$_{KpO2}$ + Al, and NP-OPS$_{KpO2}$ 7 days post-injection.
